# Physically Crosslinked Poly (Vinyl Alcohol)/Kappa-Carrageenan Hydrogels: Structure and Applications

**DOI:** 10.3390/polym12030560

**Published:** 2020-03-03

**Authors:** Catalin Croitoru, Mihai Alin Pop, Tibor Bedo, Mihaela Cosnita, Ionut Claudiu Roata, Iosif Hulka

**Affiliations:** 1 Materials Engineering and Welding Department, Transilvania University of Brasov, Eroilor 29 Str, 500036 Brasov, Romania; ionut.roata@unitbv.ro; 2 Materials Science Department, Transilvania University of Brasov, Eroilor 29 Str, 500036 Brasov, Romania; bedo.tibor@unitbv.ro; 3 Product Design Mechatronics and Environment Department, Transilvania University of Brasov, Eroilor 29 Str, 500036 Brasov, Romania; mihaela.cosnita@unitbv.ro; 4 Research Institute of renewable energy–ICER, Politehnica University of Timisoara, Piata Victoriei Str., 300006 Timisoara, Romania; hulka_iosif@yahoo.com

**Keywords:** poly (vinyl alcohol), κ-carrageenan, physical crosslinking, freeze-thaw cycles, FTIR spectroscopy, thermal analysis, sorption

## Abstract

This paper discusses the structure morphology and the thermal and swelling behavior of physically crosslinked hydrogels, obtained from applying four successive freezing–thawing cycles to poly (vinyl alcohol) blended with various amounts of κ-carrageenan. The addition of carrageenan in a weight ratio of 0.5 determines a twofold increase in the swelling degree and the early diffusion coefficients of the hydrogels when immersed in distilled water, due to a decrease in the crystallinity of the polymer matrix. The diffusion of water into the polymer matrix could be considered as a relaxation-controlled transport (anomalous diffusion). The presence of the sulfate groups determines an increased affinity of the hydrogels towards crystal violet cationic dye. A maximum physisorption capacity of up to 121.4 mg/g for this dye was attained at equilibrium.

## 1. Introduction

Poly (vinyl alcohol) (PVA) hydrogels are well known for their excellent processability [1], biocompatibility, elasticity, porosity and tunable diffusional properties [2,3], making them of prime importance in various fields, such as medicine (wound dressing, cell growth scaffolds, contact lenses and hygiene products) [4], pharmacy (controlled drug delivery vehicles) [5,6], the food industry [7], environmental applications (sorption of pollutant species and sensors) [8,9] and so forth. These materials can be obtained through various methods, which include chemical (covalent) crosslinking through radiation, the addition of mono- or bifunctional agents, or through thermal crosslinking. Numerous studies in the last three decades have focused on milder and more environmental-friendly physical crosslinking methods, among which the most promising is represented by cryostructuring (i.e., submitting the polymer to alternated freezing and thawing cycles) [10].

Many polysaccharides, in their natural or chemically functionalized state, such as chitosan, starch, dextran, cellulose, xanthan, β-glucans, sodium alginate, carrageenans and so forth, have been previously blended with PVA to obtain cryogels [11,12]. Due to their overall hydrophilic character, the addition of polysaccharides (e.g., starch, hydroxyethyl starch) can increase the swelling degree of the blend cryogels up to 300% compared with neat PVA, submitted to identical physical crosslinking operational parameters [13,14]. Chitosan, starch and various glucans, for example, are reported to improve the hydrophilic character, elasticity and flexibility of the hydrogels for wound dressing applications, while also fine-tuning the diffusion coefficient of various active principles (antibiotics, nutraceuticals and so forth) through the polymeric matrix, from 10^−5^–10^−4^ mm^2^/min with up to one order of magnitude [15,16]. 

Carrageenans (ι, λ or κ) represent a class of linear sulfated polysaccharides deriving from various species of moss, seaweed and algae. The addition of sulphated polysaccharides to PVA could modulate the release of cationic active principles from the polymer matrix in drug delivery devices [17] or could impart several functional properties to the cryogel, such as antimicrobial character or stimulatory effect for the fibroblast synthesis of collagen for tissue rebuild [18,19]. Also, the presence of the negatively charged sulfate moiety could increase the affinity of the hydrogel for cationic dyes and heavy metal ions, deeming the material useful in environmental applications [20]. 

This paper aims to obtain hydrogels comprised of PVA and κ-carrageenan (CAR) through cryostructuring (cryogelling) the aqueous solution mixtures of the two polymers by alternate freezing and thawing cycles. 

Even if PVA/carrageenan chemically-crosslinked or γ radiation-crosslinked hydrogels are already known and their suitability for controlled delivery devices [21,22,23] is under study, there are few studies regarding the obtaining of PVA/CAR cryogels, such as those by Zhang et al. [24], Hosseinzadeh [25] and Chopra et al. [26], and their use is intended either as composites with various fillers or in lyophilized form. The influence of the quantity of κ-carrageenan on the structural features (intermolecular chemical bonding, crystallinity and morphology) and the sorption/release mechanism in/from the polymer matrix has not been studied extensively up to date for this polymer blend. The sorption kinetic and adsorption mechanism assessing for a widely-employed model cationic pollutant dye, i.e., crystal violet, enables a facile comparison of the results presented in this paper with the reference literature and contributes to the enriching of the international database regarding the potential applications of hydrogel systems.

## 2. Experimental 

### 2.1. Materials

Poly (vinyl alcohol) (PVA) with M_w_~130,000, >99% hydrolyzed, was purchased from Sigma-Aldrich (Munich, Germany). The carrageenan (κ-carrageenan, CAR) with a dynamic viscosity of 15 mPa s, 0.3% in H_2_O (25 °C) and an ester sulfate group (OSO_3_^−^) percent of 20.40%, determined experimentally according to the gravimetric method proposed by Dodgson and Price [27], was purchased from Sigma-Aldrich (Munich, Germany). Both polymers were further used without any subsequent purification. 

Methylene blue (C_16_H_18_ClN_3_S), crystal violet (C_25_N_3_H_30_Cl), cyclohexane, ethanol and the inorganic reagents used in this study were purchased from the same company.

### 2.2. Gels Obtaining

The hydrogels were prepared starting from aqueous solutions of the two polymers (10% wt. for PVA, respectively 1.5% wt. for carrageenan). To prepare the aqueous PVA and carrageenan solutions, the corresponding amounts of the polymers were dissolved in distilled water at 80 °C under magnetic stirring (200 rpm) for 2 h until a transparent viscous system was obtained. Several mixtures between the two polymer solutions (10 mL of total volume) were obtained at 80 °C under magnetic stirring, as depicted in Table 1. 

The mixtures were cast in 80 mm diameter glass Petri dishes, cooled to room temperature and further submitted to four freezing–thawing cycles. The coding of the samples reflected the gravimetric ratio between carrageenan and PVA.

**Figure 1:**
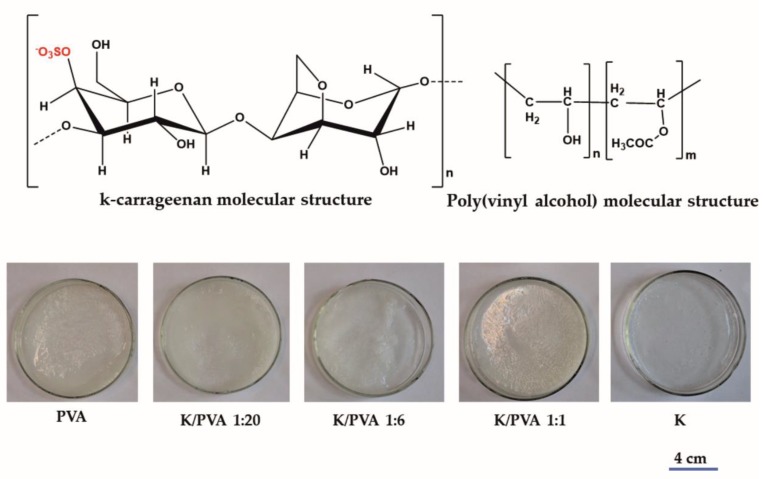
Molecular structure of the polymers and the photographic images of the obtained hydrogels.

The parameters employed for the freezing–thawing cycles were the following: freezing temperature −25 °C, freezing duration: 12 h; thawing temperature: 22 °C and thawing duration: 12 h. These parameters were the optimum ones, considering the mechanical properties of the gels (i.e., they should allow easy manipulation without rupturing), and were chosen based on our prior experience with PVA cryogels [28] or with lignocellulose-ionic liquid cryogels [29]. The aspect of the hydrogels could be seen in Figure 1.

The solids content (SC) of the gels were determined as the percentage ratio between the mass of the oven-dried gels (105 °C, 6 h) and the mass of the as-obtained gels and are depicted in Table 1.

The gel content (G) of the materials was determined as follows: the hydrogels were packed in a stainless-steel mesh (25 μm), dried until constant mass and extracted with distilled water in a Soxhlet apparatus for 5 h. After extraction, the gels were re-dried, and the gel content was calculated as the mass of the extracted dried gel, reported to the mass of the initial dried material [30].

The Shore hardness values (00 scale; PCE-OO durometer, PCE Instruments, Palm Beach, FL, USA) for the obtained gels are also illustrated in Table 1. It should be noted that for wound-dressing or drug delivery applications, the specialty literature recommends a minimum hardness value of 60 Shore 00 units [31].

All the obtained hydrogels were processed/analyzed immediately after obtaining, to avoid their structural modification with ageing. Morphology analysis (SEM), IR and XRD structural analyses require the drying of the gels. Swelling kinetic studies are performed on the as-obtained gels and for crystal violet sorption experiments, the gels are swollen (equilibrated) in distilled water, as described in Section 2.3 and Section 2.4. The bulk of the samples are stored in distilled water (in the swollen state).

### 2.3. Morphological and Structural Characterization 

A Quanta FEG 250 scanning electron microscope (FEI, Thermo Fisher Scientific, Hillsboro, OR, USA) was used to determine the morphology of the gels, at a 30 kV acceleration voltage. 

The pore volume ratio was determined by immersing the dried gels (m_dry_) in 10 mL of cyclohexane for 1 h. The cyclohexane uptake into the porous network was measured as the increase in weight of the material (m_swollen_). The pore volume ratio (*Pr*) was calculated according to Equation (1) [32]:

(1) $$Pr=\frac{m_{swollen}-m_{dry}}{m_{dry}}\cdot 100\left(\%\right).$$

For scanning electron micrography (SEM) analysis and porosity-related determinations, the gel samples were chemically dehydrated by successively immersing them in ethanol solutions of 70%, 80%, 90% and 98% vol., at 10 min for each solution, followed by drying in a desiccator for 30 min, according to the procedure described by Braet et al. [33].

The XRD spectra of the dried gel samples were acquired using a Bruker D8 diffractometer (Cu K_α_ radiation source at 0.1542 nm, Bruker, Billerica, MA, USA), scanning speed of 0.04° s^−1^ and a Bragg angle interval of 2θ = 10–50°.

Fourier-transform infrared spectroscopy (FTIR) was used to acquire the IR spectra of the dried gels in the 4000–600 cm^−1^ domain (Bruker Vertex 70 spectrophotometer, 4 cm^−1^ scanning resolution, ATR mode, 10 scans/spectrum, Bruker, Billerica, MA, USA). 

For XRD and IR analysis, the gels were dried in a P_2_O_5_-containing desiccator for 7 days. 

Differential scanning calorimetry (DSC) analysis of the as-obtained samples was performed in the [−60; +250] °C domain under a nitrogen atmosphere, with a 10 °C/min cooling/heating rate (DSC-200 F3 Maia, Netzsch, Selb, Germany).

Simultaneous thermal analysis (STA) refers to the simultaneous application of Thermogravimetry (TG) and Differential Scanning Calorimetry (DSC) to the same sample in one instrument. Analysis of the samples was performed in the [+20; +450] °C domain under a nitrogen atmosphere, with a 10 °C/min cooling/heating rate (STA 449F3 Jupiter, Netzsch, Selb, Germany).

### 2.4. Swelling and Sorption Experiments 

The swelling degree (SD, Equation (2)) of the hydrogels in distilled water, in aqueous media with the pH between 3.0 and 13.0 (namely 3.0; 5.0; 9.0 and 13.0, adjusted with solutions of 5 N HCl and NaOH, respectively), and in phosphate-buffered saline solution (PBS) (pH 7.4) at room temperature (22 °C) were determined by recurrent immersion of circular pre-weighed samples (initial mass, *m_0_*) in *V_s_* = 50 mL of fluid, followed by their extraction at pre-determined time intervals from the aqueous phase, removing excess liquid from their surface with filter paper and weighing (m_t_), until reaching of the equilibrium sorption value.

(2) $$SD=\frac{m_{t}-m_{0}}{m_{0}}\cdot 100\left(\%\right).$$

The PBS solution was obtained following the Cold Spring Harbor Protocol, by dissolving Na_2_HPO_4_ (1.4196 g), KH_2_PO_4_ (0.2449 g), NaCl (8.0066 g) and KCl (0.2012 g) in distilled water to make up 1 L of solution. 

The amount of κ-carrageenan leached from the gels during swelling was determined spectrophotometrically, based on the complexation reaction between this polymer and methylene blue (MB) dye in aqueous solutions. According to the procedure adapted from Ling and Heng [34], 100 μL of solution were withdrawn from the liquid swelling media (for the CAR gel, this aliquot of solution was prior diluted 100-fold, from which the same volume was extracted) at determined time intervals and mixed with 1000 μL of 10 mg/L MB and 900 μL of distilled water. The absorbance of the resulting solution was measured at *λ_max_* = 559 nm (Spekol 11 spectrophotometer, Carl Zeiss, Jena, Germany, glass cuvettes with a 0.996 cm optical path), and the concentration of carrageenan in the swelling media (*c_CAR_*) was determined based on the prior-constructed calibration curves (sensibility of 5 mg/L CAR, Lambert–Beer law linearity limit: 60 mg/L). The percent amount of carrageenan leached into the swelling fluid, reported to the initial amount of CAR from the gel (*f_CAR_*) was determined with Equation (3):

(3) $$f_{CAR}=\frac{c_{CAR}\cdot V_{S}\left(mL\right)}{m_{0}\cdot SC\cdot w_{CAR}}$$

where *w_CAR_* is the weight fraction of carrageenan from the obtained gels (see Table 1), c_car_ is the concentration of κ-carrageenan leached into the aqueous storing solutions (μg/mL), vs. is the volume of the swelling medium in contact with the gels (50 mL), m_0_ is the initial mass of the gel and SC is the solids content of the gels.

The crystal violet (CV) adsorption experiments were performed by immersing water-equilibrated pre-weighed (w_0_) circular-cut samples in a volume of V = 10 mL of aqueous dye solutions with initial concentrations *c_0_* = (20, 30, 40, 50, 60, 70 and 80) mg/L at 25 °C. The gels were maintained in contact with the CV solutions for 72 h, to ensure the achieving of the sorption equilibrium. The dye uptake was measured based on the decrease in the optical absorbance of the solutions, measured at *λ_max_* = 590 nm, taking into consideration the calibration curves for this dye. The equilibrium adsorption capacity of the gels (Q_e_) was calculated with Equation (4),

(4) $$Q_{e}=\frac{\left(c_{0}-c_{eq}\right)\cdot V\left(L\right)}{w_{0}\cdot SC}\cdot 100(\mathrm{mg}/g)$$

where *c_eq_* is the equilibrium concentration of the dye solutions (mg/L) after sorption equilibrium attainment.

To assess the spontaneity of the adsorption process, several thermodynamic potentials, such as the free energy (ΔG°), enthalpy (ΔH°) and entropy (ΔS°), have been determined from the van’t Hoff plots, by representing the sorption equilibrium constant K_e_ (K_e_ = Q_e_/c_e_, for c_0_ = 80 mg/L) as a function of reciprocal temperature (T: 22 °C; 25 °C; 28 °C, 30 °C and 32 °C), according to Equations (5) and (6) (R: universal gas constant, 8.31 J/mol K) [35,36]:

(5) $$logK_{e}=\frac{\Delta S{}^{\circ}}{2.303R}-\frac{\Delta H{}^{\circ}}{2.303\cdot RT}$$

(6) ΔG°=ΔH°−TΔS°.

The sorption kinetics of the CV in the gels was studied by determining the variation in the dye uptake value, *Q_t_* (mg/g), through measuring the concentration of the dye at different time intervals (*c_t_*) until the reaching of the sorption equilibrium, using the same volume of dye, i.e., 10 mL. The values of *Q_t_* were calculated with Equation (4), by replacing *c_eq_* with *c_t_.* For determining the sorption kinetic, an initial concentration of *c_0_* = 80 mg/L CV was used.

For the swelling and sorption studies, each experiment was performed in triplicate, and the average value was presented in the “Results and Discussion” Section.

## 3. Results and Discussion

### 3.1. Gels Structure and Morphology

The cryogelation process of PVA implies phase separation during freezing, which leads to the formation of a water-rich phase and a polymer-rich phase. The poly (vinyl alcohol)/κ-carrageenan/water ternary mixtures could be formed beside the water-rich phase of two polymer rich-phases, one containing a blend of the two polymers and one containing κ-carrageenan, which could separate from the blend at high concentrations [37,38]. The polymer-rich phase is mainly composed of amorphous domains interconnected with several crystalline domains (i.e., crystallites) where the macromolecular chains come in close contact with each other and interact through hydrogen bonding (i.e., cryostructuring). The crystallites represent, in fact the crosslinks responsible for the formation of the cryogels macromolecular network [11]. 

**Figure 2:**
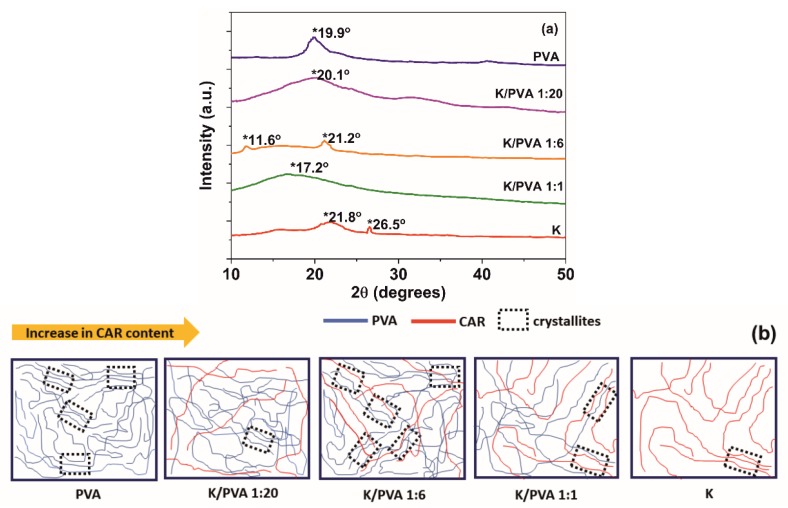
(**a**): XRD diffractograms of the obtained hydrogels; (**b**): a tentative mechanism for hydrogels cryostructuring.

The XRD diffraction pattern of PVA (Figure 2a) is composed of one broad and low-intensity diffraction hallo centered at 2θ~20° (the (101) diffraction plane of a monoclinic unit cell), which indicates a low crystallinity degree (4.2%, Table 2), following other studies regarding PVA hydrogels [39,40]. The diffractogram of pure κ-carrageenan reveals the presence of an amorphous hallo centered at 2θ = 21.8° and a weak diffraction contribution at 26.5°, which was ascribed in previous studies to inorganic trace impurities. The last peak does not appear in the diffractograms of the PVA/CAR mixtures, probably due to the dilution effect [41]. 

It can be concluded that in contrast to PVA, pure κ-carrageenan does not form crosslinked hydrogels through the application of successive freeze-thaw cycles (almost null gel content: Table 1, and low crystallinity: Table 2). However, its blending with PVA in low amounts determines a cryostructuration of the resulting gel, with the formation of an increased number of highly-ordered domains (crystallites, Figure 2b), through intermolecular cooperative hydrogen bonding between the two polymers (evidenced by FTIR spectroscopy) [42]. 

An increase in crystallinity comparing to PVA was observed for the K/PVA 1:6 gel (two diffraction peaks at 11.6° and 21.2°, the latter being ascribed to the crystallites formed by PVA). A relatively high value of crystallinity is observed for K/PVA 1:20, while at high CAR: PVA ratios, such as in the K/PVA 1:1 gel, carrageenan determines the destructuration of the crystalline nature of the material. 

Shifting of the ~20° maximum to higher diffraction angles for K: PVA 1:6 and K/PVA 1:20 implies a decrease of the crystallite size (through an increase in the macromolecular interactions by hydrogen bonding). In contrast, for K/PVA 1:1, shifting of the diffraction maximum at lower angles implies an increase in the crystallite dimensions (larger crystallites could be formed through the inclusion of CAR macromolecules). The crystallite sizes (*D*) ascribed to 2θ ~20° (Table 2) are determined with the Debye–Scherrer equation (Equation (7)) [40]:

(7) $$D=\frac{0.89\cdot \lambda }{B\cdot cos\theta }$$

where *λ* is the wavelength corresponding to the Cu Kα line (0.40784 nm), *B* is the full width at half maximum of the diffraction peak and *θ* is the diffraction angle maximum (in radians).

The number of crystalline domains in the hydrogel network plays an essential role in the performance of these materials. A high crystallinity determines a higher toughness (indicated by the higher Shore 00 hardness values from Table 1 for the PVA and K/PVA 1:6 cryogels), better dimensional stability (through the higher gel content *G*, depicted in Table 1) and lower diffusion coefficients of the active principles from the polymer network. 

**Figure 3:**
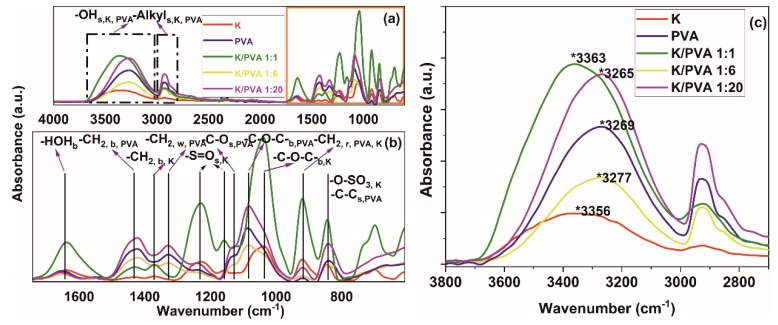
ATR-FTIR spectra of the obtained hydrogels (**a**); detail of the IR fingerprint region (**b**); detail on the 3800–2700 cm^−1^ region (**c**); spectra detail for -OH and alkyl groups region (s: stretching; b: bending; w: wagging; r: rocking vibrations).

The fingerprint region of the IR absorption spectra (Figure 3a,b) reveals typical absorption bands of PVA, occurring at 839, 917 and 1089 cm^−1^ ascribed to C–C bending, -CH_2_ rocking and ester C-O-C bending from the acetyl groups [39,43,44,45]. In κ-carrageenan, the vibrations from 841, 921 and 1035 cm^−1^ could be assigned to -C-O-SO_3_- stretching vibrations from the galactose-4-sulphate, -CH_2_ rocking from the 3,6-anhydro-D-galactose structural unit, glycosidic -C-O-C- linkage (from the 3,6 anhydro-D-galactose) and ester sulfate -S=O stretching (1159, 1235 cm^−1^) [46,47].

The vibration at 1128 cm^−1^ from PVA is ascribed to the stretching of C-O from the crystalline region of the macromolecular chain where hydrogen bonds are formed between two neighboring isotactic OH groups. The ratio between the height of the bands centered at 1128 and 1089 cm^−1^ could be regarded as a crystallinity index (*CrI*) [43,45], proportional to the crystallinity calculated from the XRD diffractograms (*Cr^XRD^*) (Table 2).

For both PVA and CAR, strong, broad absorptions are registered at 3269 cm^−1^ (PVA) and 3356 cm^−1^ (CAR), attributed to the stretching vibration ν(O-H) from hydrogen-bonded -OH groups and adsorbed water (Figure 3c). Supplementary, bending vibrations due to non-bonded water presence δ(O–H) are registered at ~1640 cm^−1^. Also, the strong absorptions at ~2932 cm^−1^ (PVA) and ~2925 cm^−1^ (CAR) are attributed to C-H stretching [43,44,45,46,47,48,49]. 

No new absorption bands apart from that characteristic of PVA, respectively CAR, could be noticed in the FTIR spectra of PVA/CAR mixtures, which could be an indication of physical interaction between the two components through hydrogen bonding. The crystallinity-sensible band of PVA (1128 cm^−1^) shifts to lower wavenumbers for the case of K/PVA 1:6 and K/PVA 1:20, indicating an increase in the intra- and intermolecular associations between the macromolecules through physical bonding (O-H…O), as found in the reference literature for other PVA-(bio)polymer mixtures [42,48]. Similarly, the -S=O stretching band at 1159 cm-^1^ shifts to lower wavenumbers and increases in width (determined at half maximum) in all PVA/CAR mixtures, possibly indicating SO_3_H…O hydrogen bonding between CAR and PVA [18]. 

The O-H…O distances (R, expressed in Å) in hydrogen-bonded crystalline domains could be correlated to the wavenumbers of infrared absorption bands attributed to the O-H stretching vibrations (ν_OH_) with the Pimentel–Sederholm equation (Equation (8)) [50]:

(8) $$\Delta \nu \left(cm^{-1}\right)=4430\cdot \left(2.84-R\right)$$

where Δν (cm^−1^) = ν_OH_- ν_0_; and ν_0_ is the wavenumber associated with free -OH groups stretching (3650 cm^−1^).

The hydrogen bonds energy (E_H_) could be calculated with Equation (9) [51]:

(9) $$E_{H}=\frac{1}{K}\cdot \frac{\nu_{0}-\nu_{OH}}{\nu_{0}}$$

where K is a constant equal to 1.6 10^−2^ kcal [51].

It can be seen from Table 2 that strong interactions through hydrogen bonding occurs for the PVA hydrogel, K/PVA 1:20 and especially K/PVA 1:6 (high values of E_H_, respectively low values for R). The crystallinity index determined through FTIR band intensity ratios is proportional to the XRD-related crystallinity.

**Figure 4:**
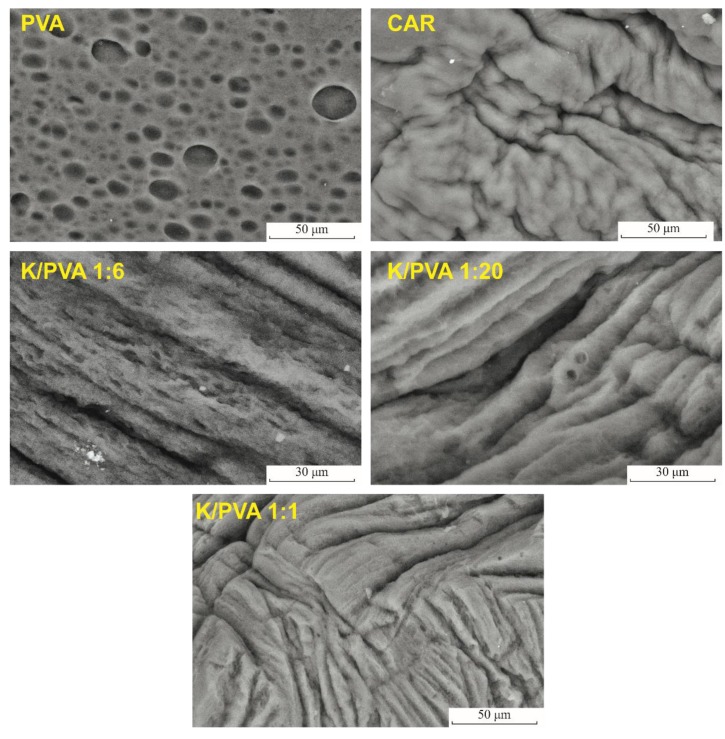
SEM micrographs of the obtained hydrogels.

Freezing of the water-rich regions of the PVA aqueous solutions determines the formation of ice crystals, which, upon thawing, generate the characteristic pore network in the structure of the cryogel. Macropores with an average diameter of 9.7 μm were observed on the surface of the dried PVA hydrogel (Figure 4).

The freeze-thawed κ-carrageenan material has a compact surface, with no apparent porosity, which indicates that this polymer’s gel does not form its pore network through the same mechanism as PVA (the water-polymer phase separation occurs in a low amount). Carrageenan addition determines a decrease in the pore size and their relative amount, as can be seen from the SEM micrographs of the dried hydrogels (Figure 4) and the cyclohexane uptake-related pore volume ratio (Table 2). In accordance with the XRD crystallinity and the FTIR crystallinity index of the polymer matrix, the highest porosity for the blend cryogels is recorded for K/PVA 1:6, as, in this case, a maximum increase in polymer-rich zones through phase separation occurs. 

Rearrangement of the polymer macromolecules and extensive intermolecular hydrogen bonding determines a compacting of the polymer phase and increase in the pore size, as it was observed in other studies [52,53]. K/PVA 1:20 and K/PVA 1:1 exhibit phase separation to a lower extent.

**Figure 5:**
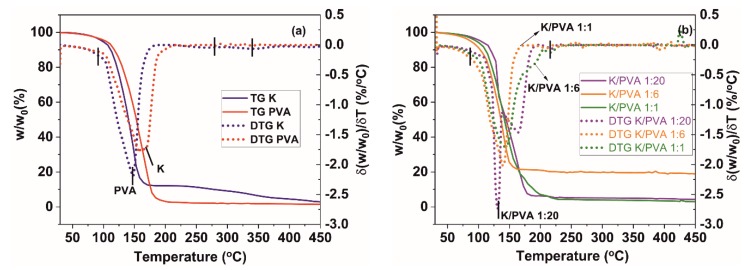
TGA and DTG thermograms of (**a**) PVA and CAR; (**b**) PVA/CAR mixtures.

The thermal behavior of PVA, CAR and PVA/CAR mixtures could be observed from the TGA and DTG (first derivative data of the TG curve) thermograms in Figure 5a,b. 

The DTG thermograms of the hydrogels reveal the presence of several thermal mass loss/decomposition stages (Table 3). The first mass loss stage is attributed to water evaporation from the materials. It can be observed that the evaporation onsets for the PVA and K materials are generally lower than those for the PVA/CAR mixtures (except for the K/PVA 1:1 hydrogel), which could indicate a higher degree of interaction between the water and polymer(s) through hydrogen bonding [54].

Degradation generally occurs in three distinct steps for all the materials. For PVA, the first degradation step involves the formation of double bonds in the polymer backbone through water elimination, the second degradation step involves the formation of aldehyde and alkene end groups, and the third corresponds to the further degradation of the remaining organic residue [55,56]. For CAR, the first degradation step involves the cleavage of the -CH-O-SO_3_^−^ bonds, secondly, of the glycosidic bonds between the 3,6 anhydro-D-galactose units, and finally degradation of the bulk polymer backbone [57]. It seems that CAR addition determines an increase in the first degradation step temperature onset, possibly due to an increase in the material’s crystallinity. An indication of the excellent compatibility between PVA and CAR (co-blending) and the mixture’s thermal stability could also be linked with the values of the mass loss due to degradation (*∆m^degrad^*, calculated as the percentual mass difference between the mass of the material at the onset of first degradation stage, and the final mass, determined at 450 °C), respectively with the values of the residue mass (*∆m^residue^*, determined at 450 °C) depicted in Table 3. These values are lower for the PVA/CAR hydrogels, compared to the PVA and K hydrogels. Remarkable stability has been registered for the K/PVA 1:6 hydrogel, similar to other blends of poly (vinyl alcohol) (e.g., PVA/β-cyclodextrin gels [58], PVA-dextran gels [59] or PVA-starch films [60]). 

Apart from the material’s crystallinity increase with the addition of CAR (0.047% and 0.139% weight ratios), thermal stability could be enhanced by the decrease in the porosity of the hydrogels (also observed in the SEM micrographs from Figure 4). Therefore, in low amounts, κ-carrageenan acts as a thermal insulator and mass transport barrier for the volatile products generated during thermal decomposition.

**Figure 6:**
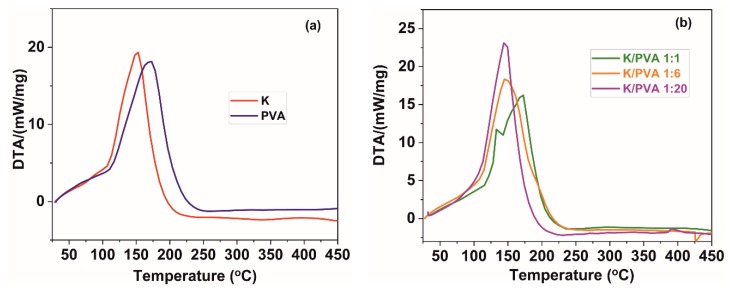
DTA thermograms of (**a**) PVA and CAR; (**b**) PVA/CAR mixtures.

Several endothermic peaks could be observed in the DTA thermograms of the PVA and K hydrogels (Figure 6a) and respectively of the PVA/CAR mixtures (Figure 6b). The broad shoulder from 95–105 °C could be ascribed to associated and non-associated water vaporization from the polymer gel. In contrast, the peaks at ~170 °C (PVA), and ~150 °C (K) could be attributed to the melting of PVA [56] and the double-helix conformation destructuration (helix to coil transition) in the case of κ-carrageenan [61]. The exact temperature values for the two transitions are depicted in Table 3. For the K/PVA 1:6 and K/PVA 1:20 hydrogels, only a single peak could be registered by DTA, which could be an indicator of an excellent compatibility between the two polymers and their reciprocal stabilization. 

**Figure 7:**
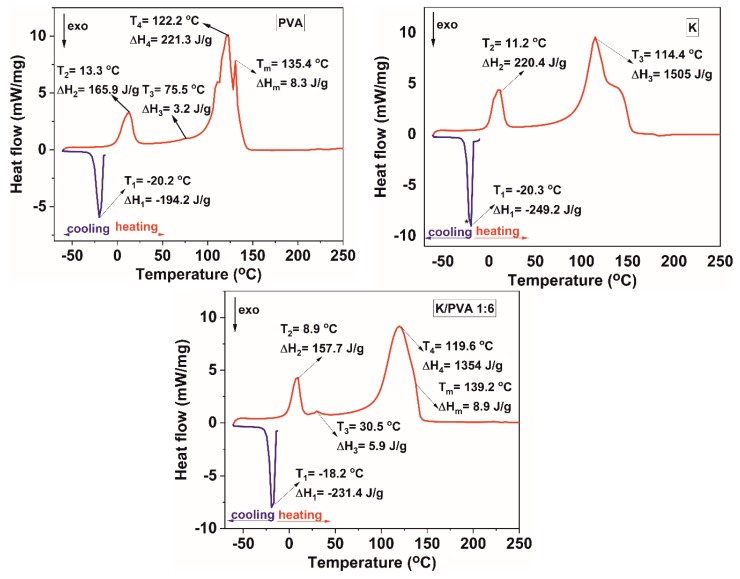
DSC thermograms of the obtained hydrogels.

The cooling curve of the thermograms presented in Figure 7 (for PVA, K and the K/PVA 1:6 hydrogel; others not shown here) contains one feature, namely a broad exothermic peak ascribed to the crystallization of water from the hydrogel network [62]. The heating profiles from the DSC thermograms illustrated in Figure 7 reveal several endotherm peaks, indicative of the multiple transitions occurring in this complex system [63]. 

An endotherm peak at ~13 °C (PVA) and ~11 °C (K) indicates the melting temperature of the non-associated (free) water in the hydrogel [62]. The temperature maxima associated with this transition for the PVA/CAR hydrogels are lower than those corresponding to PVA and K. This decrease in “thawing” temperature is proportional to the κ-carrageenan content from the gels. For the gel with the highest crystallinity degree and highest thermal stability (K/PVA 1:6), this temperature is ~9 °C, as illustrated in Figure 7. 

Generally, the presence of a single glass transition peak in the DSC thermograms for a polymeric blend can be related to good impermissibility and interaction between the constituents [42]. In this case, however, a single value for *T_g_* is not correlated to the formation of inter-polymer complexes through hydrogen bonding because κ-carrageenan does not show any presence of this type of transition. For the PVA hydrogel, a peak at ~75 °C could be ascribed to the glass transition of poly (vinyl alcohol), following the values from the reference literature [64]. 

The crystallinity of the PVA matrix in the gels (Cr^DSC^) was assessed also based on the DSC data, by dividing the value of the melting enthalpy (*∆H_m_*) to the value of the thermodynamic enthalpy corresponding to the melting of a 100% crystalline PVA (150 J/g), as reported in the literature [64]. The results, presented in Table 3 follow the same trend as the XRD-calculated crystallinity and the FTIR crystallinity index, confirming the semi-crystalline nature of the polymer matrix [40]. The highest value being registered for the K/PVA 1:6 cryogel, in which a maximum amount of phase separation and hydrogen bonding occurs. 

A decrease in the *T_g_* values was noted for all PVA/CAR mixtures in comparison with the PVA hydrogel (for the K/PVA 1:6 hydrogel, *T_g_* is ~30 °C), this decrease occurs due to the combined plasticizing effect of κ-carrageenan and water. 

Complex, broad endotherm peaks appear in the 100–150 °C temperature interval, which could be ascribed to water vaporization from PVA and K, the melting of PVA (which occurs over a range of temperatures, due to the presence of both amorphous and crystalline regions, as well as polymer chains with different stereoisomerism) and destructuration of k-carrageenan [62,63,65]. 

### 3.2. Gels Swelling and Adsorption Behavior

**Figure 8:**
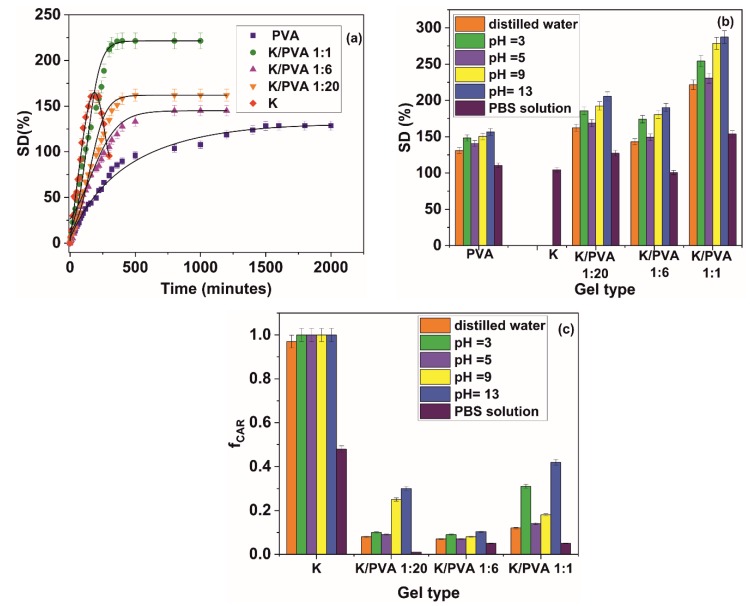
(**a**): Swelling kinetics in distilled water; (**b**): swelling degree at equilibrium in different aqueous media and (**c**): the fractional release of carrageenan at equilibrium for the obtained hydrogels.

The distilled water swelling kinetic for the obtained hydrogels is presented in Figure 8a, and the values of the swelling degree at equilibrium in different aqueous media is presented in Figure 8b.

The material comprised solely of κ-carrageenan is not stable in distilled water and aqueous media with various acidic, respectively basic pH values. This could be due to the absent crosslinking points in K, also evidenced through XRD and FTIR spectroscopy results and nil gel content value. In the PBS solution, carrageenan is more stable, probably due to the crosslinking effect promoted by the K^+^ and Na^+^ cations [66]. Increasing the κ-carrageenan content in the hydrogel determines an increase in the rate of sorption and the equilibrium water content, following the values presented in the reference literature for other types of PVA-hydrophilic biopolymer (starch, chitosan and gelatin) hydrogels [67,68,69]. 

Modification in the pH values of the solution determines an overall increase in the swelling degree of the hydrogel. Even if PVA is a non-ionic polymer, modifications in the pH could affect the swelling degree of the hydrogel due to the kosmotropic effect of the H^+^ and OH^−^ ions, which determine a restructuration of the hydrogen bonding in PVA and a modification in the association degree of bonded water molecules in the hydrogel [70]. This mechanism is also prevalent in κ-carrageenan, for which the sulfate groups are completely ionized at pH values greater than 2.8 [71]. At pH values between 5.0 and 7.00, the materials containing carrageenan are reasonably stable, as could be deducted from the fractional released amount values presented in Figure 8c. In contrast, at different pH values the carrageenan sol is disrupted, which translates into a decrease in the stability of the materials containing this type of polymer at pH values lower than 5.0 and higher than 7.0. The most stable material in terms of carrageenan leaching from the hydrogel structure is K/PVA 1:6, for which the highest crystallinity values were registered. 

Several parameters regarding the water diffusion into the hydrogel matrix were calculated, starting from the experimental swelling curves of the materials and depicted in Table 4.

The rates at which the solvent front advances from the surface to the center of the hydrogel samples were determined by calculating the media penetration velocity (ν), according to Equation (10) [72].

(10) $$v=\frac{1}{2\rho A}\cdot \frac{\partial m}{\partial t}.$$

In Equation (10), *ρ* represents the density of the swelling medium, *A* is the area of the hydrogel disc sample, and ∂m/∂t represents the time variation of the hydrogel mass when in contact with the swelling medium, calculated for *t* < 20 min, for which ∂m/∂t is constant. 

For *m_t_*/*m_eq_* ≤ 0.5, Equation (11) permits the determination of the so-called “early-time” diffusion coefficient *D_e_*, while Equation (12), valid for *m_t_*/*m_eq_* > 0.6, permits the determination of the “late-time” diffusion coefficient, *D_l_* [73]. A complementary modeling method for the early-time approximation is the power-law approach introduced by Peppas and depicted in Equation (13), where *k* is a constant indicating the swelling rate and *n* denotes the type of transport mechanism. When *n* is equal to 0.5 (and according to some researchers ≤ 0.5), the transport rate of the solute into the hydrogel is Fickian, i.e., the solvent penetration rate is higher than the polymer chain relaxation rate (Equation (12) assumes a Fickian diffusion, i.e., *n* = 0.5 and $k=4\sqrt{D_{e}}/\delta \sqrt{\pi }$). When *n* takes values between 0.5 and 1.0, the solute transport is controlled by polymer relaxation and swelling (anomalous diffusion). Case-II type diffusion occurs when *n* is equal to 1.0, while super-case II transport occurs when *n* is greater than 1.0, which both indicate to a great extent a relaxation-controlled transport [74].

(11) $$\frac{m_{t}}{m_{eq}}=4{\left[\frac{D_{e}\cdot t}{\pi \cdot \delta^{2}}\right]}^{0.5}$$

(12) $$\frac{m_{t}}{m_{eq}}=1-\frac{8}{\pi^{2}}\cdot exp\left[\frac{-D_{l}\cdot \pi \cdot t}{\delta^{2}}\right]$$

(13) $$\frac{m_{t}}{m_{eq}}=k\cdot t^{n}.$$

As can be seen from Table 4, the water penetration velocity ***ν*** in the hydrogels submerged in distilled water increases with the amount of κ-carrageenan, with one exception: namely K/PVA 1:6, for which the highest crystallinity was registered. In the latter case, the increased number of crosslink points leads to a retarding of the water penetration front into the material. With the modification of pH, all values related to the solvent penetration velocity (and implicitly polymer chain relaxation) increase. In PBS, due to the higher ionic strength of this medium and kosmotropic nature of the ions, a “collapsing” of the hydrogel network could be registered (Figure 8b), which retards the water penetration. The hydrogels exhibiting higher media penetration velocities (i.e., permeability) also presented higher values for the equilibrium swelling degree.

The early-time and late-time diffusion coefficients calculated from Equations (12) and (13) reported in Table 4 range from 5.42 10^−5^ to 8.97 10^−4^ mm^2^/min, respectively 0.0879 to 0.480 mm^2^/min, increasing in the same manner as the solvent penetration rate ν. The relatively high values for the diffusion coefficients are comparable to other PVA-containing hydrogels [75,76], and imply a high degree of polymer chains relaxation (this could also be deducted from the values of *n*, which range from 0.73 to 1.00). The hydrogels immersed in PBS represent an exception, in the sense that the water sorption is combined with the PVA “collapsing” and κ-carrageenan crosslinking. This retards the swelling of the polymer matrix. Thus, the diffusion of water in these materials follow a mechanism closer to the ideal Fickian one.

**Figure 9:**
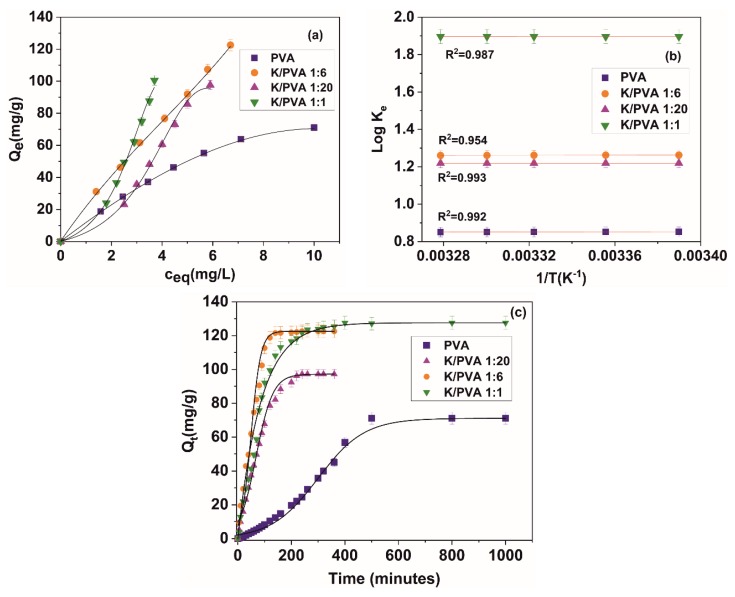
(**a**): Adsorption isotherms for CV onto the PVA/CAR hydrogels at 25 °C; (**b**): van’t Hoff plots and (**c**): kinetic for the CV sorption in the PVA/CAR hydrogels at 25 °C.

The ability of the hydrogels to function as sorbents for potentially toxic chemical species removal from wastewaters was also studied. The adsorption and kinetic mechanism for crystal violet sorption onto the previously water-equilibrated hydrogels was studied by modelling the adsorption data from Figure 9a with various isotherm models, presented in Table 5. The theory behind each type of isotherm model can be found in the reference literature [77,78,79,80]. Only the physical meaning and measurement units for each parameter were given at the end of the Table 5. Crystal violet was chosen as a model dye for sorption as it does not interact with the hydrogel components through forming complexes that have a maximum absorption at different wavelengths than the original dye and due to the vast available literature for different sorbent types and sorption conditions, allowing for easy comparison of the obtained sorption capacities. The maximum sorption capacities at equilibrium (Q_e_) for CV range from 63.2 mg/g (in the case of PVA) to 121.4 mg/g (in the case of K/PVA 1:6). 

These values are comparable to other results reported in the reference literature, for CV sorption on other types of hydrogels: 49.0 mg/g (poly (acrylic acid) hydrogels) [81]; 105.2 mg/g (carrageenan/multi-walled carbon nanotube hybrid hydrogel) [82] and 16.3 mg/g (κ-carrageenan hydrogel beads) [83].

The results from Table 5 indicate that the Freundlich and the Redlich–Peterson adsorption isotherms best fit the experimental results in the case of PVA, K/PVA 1:20 and K/PVA 1:6. This is indicative of a heterogenous non-ideal adsorption mechanism, not restricted to the formation of a monolayer of adsorbate [77]. For the K/PVA 1:20 and the K/PVA 1:1 hydrogel, the 1/n_F_ values are higher than 1, which indicate a cooperative adsorption mechanism. In the case of PVA and K/PVA 1:6, the adsorption is strongly favored. 

From Figure 9b and Table 5 we can see that the adsorption of CV onto the surface of the hydrogel is an exothermic process for PVA and K/PVA 1:6. For these two types of hydrogel, the adsorption is spontaneous and of a physical nature, indicated by the slightly negative values of ∆G [81], and is observed to decrease with the increase in temperature. For all hydrogels, the ∆S values are positive, indicating the increased randomness at the solid/liquid interface in the adsorption process. For K/PVA 1:20 and K/PVA 1:1, the adsorption is not thermodynamically favored (∆G > 0), but at higher temperatures, lower values could be registered for ∆G. 

The contact time data from Figure 9c was fitted against various kinetic models, such as pseudo-first order, pseudo-second-order, intra-particle diffusion, liquid film diffusion and Elovich. The significance of each model was not presented here; however, it can be consulted from the reference literature [84,85,86]. The equations of the kinetic models, the parameters of the equations and the determination coefficient indicating the goodness of the fit (R^2^) are depicted in Table 6. 

For PVA, K/PVA 1:20 and K/PVA 1:6, the model that best fits the experimental kinetic data is the pseudo-second order over the entire contact time range, which is consistent with the findings from the reference literature for this type of dye and hydrogel sorbents [85]. The high determination coefficients for the pseudo-second-order model implies that the sharing or exchange of electrons between the polymer substrate and CV (for PVA) and the electrostatic interaction between the ionized chemical groups of κ-carrageenan and the organic cation of CV is the rate-controlling step. For the K/PVA 1:1 hydrogel, the boundary layer effect (i.e., transport of the CV from the boundary liquid film to the surface of the adsorbing material) is the rate-limiting step. 

Charge carrier transfer, electrostatic interaction and liquid film diffusion are not the only processes involved in CV adsorption. The Weber and Morris intra-particle diffusion model fits well the first 20% of the sorption kinetic for all types of hydrogel. Based on this model, the sorption of CV on the hydrogels could be described as a three-step process: (1) the transport of CV from the boundary liquid film to the surface of the adsorbent (external film mass transfer); (2) the further transfer of CV from the surface of the adsorbate to the internal active binding sites (intraparticle diffusion); and (3) the interaction of CV with the active binding sites. The appropriateness of the Weber and Morris intra-particle diffusion model to describe the sorption kinetic in the early-time approximation is extensively described by other studies involving hydrogel or biomass-derived materials [85,86]. At longer contact times, the external mass transfer and dynamic equilibrium processes could also play an important role in CV sorption.

From the values of *k_2_* (Table 6), with the increase in the amount of κ-carrageenan, the sorption process occurs with a higher rate and the equilibrium is reached in a shorter period.

## 4. Conclusions

The addition of κ-carrageenan to PVA determines the obtaining of hydrophilic hydrogels with a high distilled water swelling degree, up to 224%, compared to neat PVA, for which a value of 115% was registered. Carrageenan also determines the decrease with up to 58% in the crystallinity index for the polymer blend matrix, compared to PVA. Also, a higher water diffusion coefficient in/through the polymer matrix (up to 8.24 × 10^−4^ mm^2^/min, compared to 1.82 × 10^−5^ mm^2^/min for PVA) was attained. 

The results indicate that several types of interactions occur between the two polymers, implying hydrogen bonding. These interactions are enhanced for the hydrogel containing a κ-carrageenan weight fraction of 0.139, for which the highest stability in aqueous media of various ionic strength and pH was observed. κ-carrageenan also determines an increased affinity of the hydrogels towards crystal violet cationic dye. This physisorption process is generally thermodynamically favored and was well modelled by the Freundlich isotherm mechanism.

## Figures and Tables

**Table 1:** Characteristics of the obtained hydrogels. Poly (vinyl alcohol) (PVA), κ-carrageenan (CAR).

Sample Code	PVA		CAR		w_car_	Gels SC* (%)	Shore 00 Hardness *	Thickness * δ (mm)	G (%)
	Vol. (mL)	Amount (g)	Vol. (mL)	Amount (g)					
PVA	10	1	-	-	0	9.85	68	2.85	82.24
K/PVA 1:20	7.5	0.75	2.5	0.037	0.047	7.59	64	2.94	76.15
K/PVA 1:6	4.8	0.48	5.2	0.078	0.139	5.98	69	3.02	83.07
K/PVA 1:1	1.3	0.13	8.7	0.13	0.5	2.62	45	3.11	64.80
K	-	-	10	0.15	1	1.54	12	3.16	0.75

* Maximum relative error: for SC and G: ± 1.5%, for the thickness: ± 0.1%, and for the Shore hardness: ± 0.5%. w_car_: weight ratio of carrageenan; SC: solids content (%); G: gel content (%).

**Table 2:** The characteristics of the obtained hydrogels.

Sample Code	FTIR			XRD		Pore volume Ratio Pr (%)
	CrI	R (Å)	E_H_ (kcal)	Cr^XRD^ (%)	D^XRD^ (nm)	
PVA	0.143	2.926	6.523	4.2	5.79	8.63
K/PVA 1:20	0.119	2.927	6.386	4.0	5.05	6.42
K/PVA 1:6	0.188	2.920	6.592	4.8	4.81	5.53
K/PVA 1:1	0.024	2.924	4.914	2.3	6.02	3.48
K	-	2.929	5.034	1.1	-	2.31

**Table 3:** The thermal properties of the PVA/CAR hydrogels according to the TGA, DTA and DSC thermogram results.

Sample Code	TGA/DTG Results			DTA Results		DSC Results	
	T^water^ (°C)	T^degrad^ (°C)	∆m^degrad^ (%)	∆m^residue^ (%)	T^DTA^ (°C)	T_g_ (°C)	Cr^DSC^ (%)
**PVA**	100.2	146.5 273.5 340.3	95.5	4.0	170.7	75.5	5.5
**K/PVA 1:20**	109.2	133.6 167.1 225.5	91.4	4.6	132.7 169.7	60.5	5.2
**K/PVA 1:6**	113.1	149.1 185.9 214.7	37.8	19.4	145.8	30.5	5.9
**K/PVA 1:1**	104.0	145.7 206.7 268.8	90.7	3.5	145.5	29.5	3.2
**K**	106.8	176.8 288.1 333.6	91.9	1.2	150.4	-	-

**Table 4:** Parameters regarding the diffusion of water into the hydrogel matrix.

Material	Parameter	Swelling Medium					
		Distilled Water	pH = 3	pH = 5	pH = 9	pH = 13	PBS
**PVA**	***ν* (cm/min × 10^3^)**	1.82	1.89	1.84	1.86	2.11	1.67
	***D_e_* (mm^2^/min × 10^5^)**	1.82 (0.992)	1.94 (0.984)	1.90 (0.990)	1.84 (0.994)	2.01 (0.982)	1.43 (0.992)
	***D_l_* (mm^2^/min × 10^2^)**	6.72(0.977)	8.92 (0.971)	8.87 (0.989)	8.79 (0.977)	8.94 (0.975)	5.11 (0.976)
	***k* (min^−0.5^ × 10^3^)**	4.10 (0.964)	4.89 (0.960)	5.16 (0.976)	4.63 (0.983)	4.75 (0.974)	3.46 (0.988)
	***n***	0.73 (0.964)	0.85 (0.960)	0.82 (0.976)	0.84 (0.983)	0.91 (0.974)	0.68 (0.988)
**K/PVA 1:20**	***ν* (cm/min × 10^3^)**	1.89	1.52	0.93	0.95	1.61	0.72
	***D_e_* (mm^2^/min × 10^5^)**	6.31 (0.968)	6.58 (0.972)	6.40 (0.992)	6.61 (0.988)	6.72 (0.988)	5.62 (0.995)
	***D_l_* (mm^2^/min × 10^1^)**	2.30 (0.979)	2.65 (0.989)	2.40 (0.995)	2.69 (0.985)	2.73 (0.992)	1.95 (0.984)
	***k* (min^−0.5^ × 10^3^)**	1.96 (0.967)	1.99 (0.973)	1.97 (0.989)	2.01 (0.9)	4.75 (0.974)	3.46 (0.988)
	***n***	0.99 (0.967)	0.85 (0.960)	0.82 (0.976)	0.84 (0.983)	0.91 (0.974)	0.68 (0.988)
**K/PVA 1:6**	***ν* (cm/min × 10^3^)**	1.65	1.78	1.69	1.71	1.82	1.38
	***D_e_* (mm^2^/min × 10^5^)**	6.66 (0.986)	7.80 (0.989)	6.75 (0.992)	6.72 (0.994)	7.84 (0.992)	5.42 (0.991)
	***D_l_* (mm^2^/min × 10^1^)**	2.97 (0.988)	3.18 (0.978)	3.08 (0.977)	3.11 (0.976)	3.24 (0.972)	1.47 (0.973)
	***k* (min^−0.5^ × 10^3^)**	4.0 (0.980)	4.92 (0.991)	4.27 (0.993)	4.20 (0.988)	5.02 (0.987)	3.20 (0.988)
	***n***	0.93 (0.980)	0.96 (0.991)	0.94 (0.993)	0.96 (0.988)	1.00 (0.987)	0.71 (0.988)
**K/PVA 1:1**	***ν* (cm/min × 10^3^)**	3.66	3.90	3.69	3.73	3.98	1.89
	***D_e_* (mm^2^/min × 10^4^)**	8.24 (0.993)	8.97 (0.998)	8.28 (0.997)	8.27 (0.991)	8.89 (0.992)	5.43 (0.992)
	***D_l_* (mm^2^/min × 10^1^)**	3.95(0.970)	4.72 (0.975)	4.12 (0.970)	4.06 (0.978)	4.80 (0.980)	2.99 (0.989)
	***k* (min^−0.5^ × 10^3^)**	5.23 (0.995)	5.39 (0.994)	5.28 (0.989)	5.23 (0.995)	5.23 (0.995)	5.23 (0.988)
	***n***	0.95 (0.995)	0.97 (0.994)	0.96 (0.989)	0.95 (0.995)	0.95 (0.995)	0.62 (0.988)

**Table 5:** Parameters of the isotherm models fitted onto the experimental CV adsorption data at 25 °C.

Isotherm Model	Isotherm Equation (Linearized Form)	Equation Parameters			
		PVA	K/PVA 1:20	K/PVA 1:6	K/PVA 1:1
*Langmuir*	$\frac{c_{eq}}{Q_{e}}=\frac{1}{K_{L}\cdot Q_{m}}+\frac{c_{eq}}{Q_{m}}$ $R_{L}=\frac{1}{\left(1+K_{L}\cdot c_{0}\right)}$	*K_L_* = 0.092 *Q_m_* = 153.84 *R_L_*: 0.11 ÷ 0.35 R^2^ = 0.953	*K_L_* = 0.103 Q_m_ = 76.92 *R_L_*: 0.11 ÷ 0.32 R^2^ = 0.731	*K_L_* = 0.035 Q_m_ = 625.11 *R_L_*: 0.26 ÷ 0.58 R^2^ = 0.789	*K_L_* = 0.176 Q_m_ = 55.55 *R_L_*: 0.06 ÷ 0.22 R^2^ = 0.909
*Freundlich*	$\ln Q_{e}=\ln K_{F}+\frac{1}{n_{F}}\ln c_{eq}$	*K_F_* = 14.270 *1/n_F_* = 0.77 R^2^ = 0.979	*K_F_* = 5.371 *1/n_F_* = 1.70 R^2^ = 0.976	*K_F_* = 22.510 *1/n_F_* = 0.88 R^2^ = 0.998	*K_F_* = 7.882 *1/n_F_* = 1.94 R^2^ = 0.997
*Radke–Prausnitz*	$\frac{c_{eq}}{Q_{e}}=\frac{1}{K_{RP}\cdot Q_{m}}+\frac{c_{eq}^{m}}{Q_{m}}$	*K_RP_* = 0.146 Q_m_ = 79.42 *m* = 0.65 R^2^ = 0.970	*K_RP_* = 0.763 Q_m_ = 92.90 *m* = 1.50 R^2^ = 0.967	*K_RP_* = 0.111 Q_m_ = 123.97 *m* = 0.90 R^2^ = 0.998	*K_RP_* = 0.940 Q_m_ = 112.59 *m* = 2.00 R^2^ = 0.995
*Redlich–Peterson*	$\ln\left(K_{R}\cdot \frac{c_{eq}}{Q_{e}}-1\right)=g\cdot \ln c_{eq}+\ln\alpha_{R}$ $Q_{m}=\frac{K_{R}}{\alpha_{R}}$	*K_R_* = 8.372 g = 0.74 *α_R_* = 0.11 Q_m_ = 76.10 R^2^ = 0.979	*K_R_* = 1.372 g = 0.79 *α_R_* = 0.013 Q_m_ = 99.91 R^2^ = 0.979	*K_R_* = 1.619 g = 0.81 *α_R_* = 0.013 Q_m_ = 124.59 R^2^ = 0.998	*K_R_* = 1.631 g = 0.86 *α_R_* = 0.014 Q_m_ = 116.54 R^2^ = 0.909
*Dubinin–Radushkevich*	$\ln Q_{e}=\ln Q_{m}-\beta \varepsilon^{2}$ $\varepsilon =RT\ln\left(1+\frac{1}{c_{eq}}\right)$ $E=\frac{1}{\sqrt{2\beta }}$	*Q_m_ =* 63.10 *β*: = 9 10^−7^ *E* = 745 R^2^ = 0.914	*Q_m_ =* 142.25 *β*: = 3 10^−6^ *E* = 408 R^2^ = 0.995	*Q_m_ =* 130.08 *β*: = 8 10^−7^ *E* = 791 R^2^ = 0.865	*Q_m_ =* 112.44 *β*: = 2 10^−6^ *E* = 500 R^2^ = 0.992
*Flory–Huggins*	$\ln\left(\frac{\theta }{c_{0}}\right)=\ln K_{FH}+n\cdot \ln\left(1-\theta \right)$ $\theta =1-\frac{c_{eq}}{c_{0}}$ $\Delta G{}^{\circ}=RT\cdot \ln K_{FH}$	*K_FH_* = 2.2 10^−5^ *n* = 2.85 *∆G°* = −26.48 R^2^ = 0.774	*K_FH_* = 5.378 *n* = 2.27 *∆G°* = 4.16 R^2^ = 0.951	*K_FH_* = 1.8 10^−10^ *n* = 1.90 *∆G°* = −55.44 R^2^ = 0.879	*K_FH_* = 6.529 *n* = 2.05 *∆G°* = 4.65 R^2^ = 0.996
*−*	Thermodynamic parameters according to Equations (5) and (6) and Figure 9b for c_0_ = 80 mg/L (at 25 °C)	*∆H°* = −6.7 *∆S°* = 15.6 *∆G°* = −11.3	*∆H* = 10.5 *∆S°* = 23.4 *∆G°* = 11.1	*∆H°* = −14.5 *∆S°* = 22.0 *∆G°* = −21.0	*∆H°* = 19.7 *∆S°* = 36.5 *∆G°* = 20.7

*Q_m_*: maximum adsorption capacity at monolayer coverage (mg/g); *K_L_*: Langmuir isotherm constant (L/mg); *R_L_*: Langmuir separation factor; c_0_: initial concentration of CV (mg/L); *K_F_*: Freundlich isotherm constant ((mg/g) (mg/L)^1/n^); *n_F_*: energetic heterogeneity of the adsorption sites; *K_RP_*: Radke–Prausnitz equilibrium constant (L/mg); *m*: Radke–Prausnitz model exponent; *K_R_*: Redlich–Peterson isotherm constant (L/g); *g* and *α_R_* (L/mg) are the Redlich–Peterson constants; *β*: Dubinin–Radushkevich constant (mol^2^/J^2^); *ε*: Polanyi potential (J/mol); R: universal gas constant (8.31 J/mol K); *T*: adsorption temperature (298 K); *E*: mean adsorption energy (J/mol); *θ*: degree of surface coverage; *K_FH_*: Flory–Huggins equilibrium constant (L/mg); *n*: number of adsorbates occupying the adsorption sites; *∆G°*: standard Gibbs free energy change (kJ/mol); *∆HG°:* standard enthalpy (kJ/mol); *∆S°*: standard entropy (J/mol K); R^2^: determination coefficient indicating the goodness of the fit with the proposed isotherm model [77,78,79,80].

**Table 6:** Parameters of the isotherm models fitted onto the experimental CV adsorption data.

*Kinetic Model*	*Model Equation (Linearized Form)*	*Equation Parameters*			
		PVA	K/PVA 1:20	K/PVA 1:6	K/PVA 1:1
*Pseudo-first-order*	$\log(Q_{e}-Q_{t})=\log Q_{e}-\frac{k_{1}t}{2.303}$	k_1_ = 1.8 × 10^−3^ R^2^ = 0.920	k_1_ = 6.3 × 10^−3^ R^2^ = 0.899	k_1_ = 0.018 R^2^ = 0.839	k_1_ = 9.1 × 10^−3^ R^2^ = 0.849
*Pseudo-second-order*	$\frac{t}{Q_{t}}=\frac{1}{k_{2}\cdot Q_{e}^{2}}+\frac{t}{Q_{e}}$	k_2_ = 0.4 × 10^−5^ R^2^ = 0.956	k_2_ = 1.1 × 10^−4^ R^2^ = 0.961	k_2_ = 7.5 × 10^−4^ R^2^ = 0.974	k_2_ = 9.4 × 10^−3^ R^2^ = 0.908
*Intraparticle diffusion*	$Q_{t}=k_{d}\cdot t^{1/2}+C$ (20% of the first portion of the kinetic data)	k_d_ = 0.144 C = 3.9 × 10^−8^ R^2^ = 1	k_d_ = 2.314 C = 3.1 × 10^−12^ R^2^ = 1	k_d_ = 2.354 C = 0.254 R^2^ = 1	k_d_ = 1.739 C = 3.6 × 10^−16^ R^2^ = 1
*Liquid film diffusion*	$\ln\left(1-\frac{Q_{t}}{Q_{e}}\right)=-k_{fd}\cdot t$	k_fd_ = 2.1 × 10^−3^ R^2^ = 0.891	k_fd_ = 1.5 × 10^−2^ R^2^ = 0.901	k_fd_ = 2.6 × 10^−2^ R^2^ = 0.924	k_fd_ = 1.2 × 10^−2^ R^2^ = 0.973
*Elovich*	$Q_{t}=\frac{1}{\beta }\ln(\alpha \cdot \beta )+\frac{1}{\beta }\ln t$	α = 2.1 × 10^−17^ β = 2.1 × 10^−3^ R^2^ = 0.634	α = 1.2 × 10^−8^ β = 5.8 × 10^−2^ R^2^ = 0.325	α = 6 × 10^−7^ β = 0.199 R^2^ = 0.251	α = 9.1 × 10^−4^ β = 2.9 × 10^−2^ R^2^ = 0.929

*k_1_*: rate constant of pseudo-first order kinetic (min^−1^), *k_2_*: second-order rate constant (g/mg min), *k_d_*: rate constant of intraparticle diffusion (mg/g min^1/2^), *C*: intercept of intraparticle diffusion, *k_fd_*: liquid film diffusion rate coefficients (min^−1^), *α*: (mg/g min) and *β*: (g/mg) are the initial adsorption rate of the Elovich equation, respectively the desorption constant related to the extent of surface coverage and activation energy constant for sorption [85,86].

## References

[1] Wang X.D., Liu X.H., Yuan H.Y., Liu H., Liu C.T., Li T.X., Yan C., Yan X.R., Shen C.Y., Guo Z.H. (2018). Non-covalently functionalized graphene strengthened poly(vinyl alcohol). Mater. Des..

[2] Ahmed E.M. (2015). Hydrogel: Preparation, characterization, and applications: A review. J. Adv. Res..

[3] Wang Y.J., Zhang X.N., Song Y.H., Zhao Y.P., Chen L., Su F.M., Li L.B., Wu Z.L., Zheng Q. (2019). Ultrastiff and Tough Supramolecular Hydrogels with a Dense and Robust Hydrogen Bond Network. Chem. Mater..

[4] Wang X., Su M., Liu C., Shen C., Liu X. (2020). Poly (vinyl alcohol)/Graphene Nanocomposite Hydrogel Scaffolds for Control of Cell Adhesion. J. Renew. Mater..

[5] Narayanaswamy R., Torchilin V.P. (2019). Hydrogels and Their Applications in Targeted Drug Delivery. Molecules.

[6] Ghanaatian E., Entezam M. (2019). Mechanical properties and drug release rate of poly(vinyl alcohol)/poly(ethylene glycol)/clay nanocomposite hydrogels: Correlation with structure and physical properties. J. Appl. Polym. Sci..

[7] Liu L.S., Kost J., Yan F., Spiro R.C. (2012). Hydrogels from Biopolymer Hybrid for Biomedical, Food, and Functional Food Applications. Polymers (Basel).

[8] El Halah A., Lopez-Carrasquero F., Contreras J. (2018). Applications of hydrogels in the adsorption of metallic ions. Cienc. Ing..

[9] Guenther M., Gerlach G., Sorber J., Suchaneck G., Arndt K.F., Richter A. (2005). pH sensors based on polyelectrolytic hydrogels. Smart Struct. Mater..

[10] Duquette D., Dumont M.J. (2019). Comparative studies of chemical crosslinking reactions and applications of bio-based hydrogels. Polym. Bull..

[11] Crescenzi V., Paradossi G., Desideri P., Dentini M., Cavalieri F., Amici E., Lisi R. (1997). New hydrogels based on carbohydrate and on carbohydrate-synthetic polymer networks. Polym. Gels. Netw..

[12] Sultankulov B., Berillo D., Kauanova S., Mikhalovsky S., Mikhalovska L., Saparov A. (2019). Composite Cryogel with Polyelectrolyte Complexes for Growth Factor Delivery. Pharmaceutics.

[13] Bagri L.P., Saini R.K., Bajpai A.K., Choubey R. (2019). Silver hydroxyapatite reinforced poly(vinyl alcohol)-starch cryogel nanocomposites and study of biodegradation, compressive strength and antibacterial activity. Polym. Eng. Sci..

[14] Kamoun E.A., Kenawy E.R.S., Chen X. (2017). A review on polymeric hydrogel membranes for wound dressing applications: PVA-based hydrogel dressings. J. Adv. Res..

[15] Lotfipour F., Alami-Milani M., Salatin S., Hadavi A., Jelyehgari M. (2019). Freeze-thaw-induced cross-linked PVA/chitosan for oxytetracycline-loaded wound dressing: The experimental design and optimization. Res. Pharm. Sci..

[16] Santos C., Silva C.J., Buttel Z., Guimaraes R., Pereira S.B., Tamagnini P., Zille A. (2014). Preparation and characterization of polysaccharides/PVA blend nanofibrous membranes by electrospinning method. Carbohyd. Polym..

[17] Yegappan R., Selvaprithiviraj V., Amirthalingam S., Jayakumar R. (2018). Carrageenan based hydrogels for drug delivery, tissue engineering and wound healing. Carbohyd. Polym..

[18] Bajpai S.K., Daheriya P. (2014). Kappa-Carrageenan/PVA Filmswith Antibacterial Properties: Part 1. Optimization of Preparation Conditions and Preliminary Drug Release Studies. J. Macromol. Sci. A.

[19] El-Fawal G.F., Yassin A.M., El-Deeb N.M. (2017). The Novelty in Fabrication of Poly Vinyl Alcohol/kappa-Carrageenan Hydrogel with Lactobacillus bulgaricus Extract as Anti-inflammatory Wound Dressing Agent. AAPS PharmSciTech.

[20] Soares S.F., Simoes T.R., Trindade T., Daniel-da-Silva A.L. (2017). Highly Efficient Removal of Dye from Water Using Magnetic Carrageenan/Silica Hybrid Nano-adsorbents. Water Air Soil Poll..

[21] Rasool A., Ata S., Islam A., Khan R.U. (2019). Fabrication of novel carrageenan based stimuli responsive injectable hydrogels for controlled release of cephradine. RSC Adv..

[22] Sukhlaaied W., Riyajan S.A. (2013). Synthesis and properties of carrageenan grafted copolymer with poly(vinyl alcohol). Carbohyd. Polym..

[23] Dafader N.C., Manir M.S., Alam M.F., Swapna S.P., Akter T., Huq D. (2015). Effect Of Kappa-Carrageenan On The Properties Of Poly(Vinyl Alcohol) Hydrogel Prepared By The Application Of Gamma Radiation. SOP Trans. Appl. Chem..

[24] Zhang Y.B., Ye L., Cui M., Yang B.G., Li J.J., Sun H., Yao F.L. (2015). Physically crosslinked poly(vinyl alcohol)-carrageenan composite hydrogels: Pore structure stability and cell adhesive ability. RSC Adv..

[25] Hosseinzadeh H. (2017). Freezing-Thawing Assisted Synthesis of Novel Carrageenan Nanomagnetic Beads for Controlled Release of Antitumor Drugs. Cell. Chem. Technol..

[26] Chopra P., Nayak D., Nanda A., Ashe S., Rauta P.R., Nayak B. (2016). Fabrication of poly(vinyl alcohol)-Carrageenan scaffolds for cryopreservation: Effect of composition on cell viability. Carbohyd. Polym..

[27] Dodgson K.S., Price R.G. (1962). A note on the determination of the ester sulphate content of sulphated polysaccharides. Biochem. J..

[28] Patachia S., Croitoru C. (2015). Increasing the adsorption capacity and selectivity of poly(vinyl alcohol) hydrogels by an alternative imprinting technique. J. Appl. Polym. Sci..

[29] Roata I.C., Croitoru C., Pascu A., Stanciu E.M. (2018). Characterization of Physically Crosslinked Ionic Liquid-lignocellulose Hydrogels. Bioresources.

[30] Varca G.H.C., Ferraz C.C., Mathor M.B., Lopes P.S., Rogero S.O., Rogero J.O. (2015). Encapsulation and nano-encapsulation of papain active sites to enhance radiolytic stability and decrease toxicity. Nanoscale Radiation Engineering of Advanced Materials for Potential Biomedical Applications.

[31] Kokabi M., Sirousazar M., Hassan Z.M. (2007). PVA-clay nanocomposite hydrogels for wound dressing. Eur. Polym. J..

[32] Jain E., Kumar A. (2009). Designing Supermacroporous Cryogels Based on Polyacrylonitrile and a Polyacrylamide-Chitosan Semi-interpenetrating Network. J. Biomat. Sci. Polym. Ed..

[33] Braet F., deZanger R., Wisse E. (1997). Drying cells for SEM, AFM and TEM by hexamethyldisilazane: A study on hepatic endothelial cells. J. Microsc. Oxf..

[34] Ling Y.P., Heng L.Y. Complexation between Carrageenan and Methylene Blue for Sensor Design. Proceedings of the 2013 Ukm Fst Postgraduate Colloquium.

[35] Pehlivan E., Arslan G. (2007). Removal of metal ions using lignite in aqueous solution—Low cost biosorbents. Fuel Process. Technol..

[36] Caccavo D., Lamberti G., Cafaro M.M., Barba A.A., Kazlauske J., Larsson A. (2017). Mathematical modelling of the drug release from an ensemble of coated pellets. Br. J. Pharmacol..

[37] Wang X.L., Pan Y.M., Shen C.Y., Liu C.T., Liu X.H. (2018). Facile Thermally Impacted Water-Induced Phase Separation Approach for the Fabrication of Skin-Free Thermoplastic Polyurethane Foam and Its Recyclable Counterpart for Oil-Water Separation. Macromol. Rapid Comm..

[38] Wang Y.T., Yang H.G., Chen Z.H., Chen N., Pang X.C., Zhang L., Minari T., Liu X.Y., Liu H.Z., Chen J.Z. (2018). Recyclable Oil-Absorption Foams via Secondary Phase Separation. ACS Sustain. Chem. Eng..

[39] Tretinnikov O.N., Zagorskaya S.A. (2012). Determination of the degree of crystallinity of poly(vinyl alcohol) by FTIR spectroscopy. J. Appl. Spectrosc..

[40] Ricciardi R., Auriemma F., De Rosa C., Laupretre F. (2004). X-ray diffraction analysis of poly(vinyl alcohol) hydrogels, obtained by freezing and thawing techniques. Macromolecules.

[41] Liew J.W.Y., Loh K.S., Ahmad A., Lim K.L., Daud W.R.W. (2017). Synthesis and characterization of modified kappa-carrageenan for enhanced proton conductivity as polymer electrolyte membrane. PLoS ONE.

[42] Kantoglu O., Caykara T., Guven O. (2013). Preparation and characterization of polysaccaride interpolymer complexes: I-PVA/iota-carrageenan. J. Appl. Polym. Sci..

[43] Xu W.G., Asai S., Sumita M. (1997). Spectroscopic study of ethylene vinyl alcohol copolymer and poly(vinyl alcohol). Sen’i Gakkaishi.

[44] Santos A.M.N., Moreira A.P.D., Carvalho C.W.P., Luchese R., Ribeiro E., McGuinness G.B., Mendes M.F., Oliveira R.N. (2019). Physically Cross-Linked Gels of PVA with Natural Polymers as Matrices for Manuka Honey Release in Wound-Care Applications. Materials.

[45] Mansur H.S., Sadahira C.M., Souza A.N., Mansur A.A.P. (2008). FTIR spectroscopy characterization of poly (vinyl alcohol) hydrogel with different hydrolysis degree and chemically crosslinked with glutaraldehyde. Mater. Sci. Eng. C Bio. S.

[46] Zinoun M., Cosson J., Deslandes E. (1993). Physicochemical Characterization of Carrageenan from Gigartina-Teedii (Rooth) Lamouroux (Gigartinales, Rhodophyta). J. Appl. Phycol..

[47] Pereira L., Amado A.M., Ribeiro-Claro P.J.A., van de Velde F. VIBRATIONAL SPECTROSCOPY (FTIR-ATR AND FT-RAMAN) A Rapid and Useful Tool for Phycocolloid Analysis. Proceedings of the Biodevices 2009: Proceedings of the International Conference on Biomedical Electronics and Devices.

[48] Kenawy E., Kamoun E.A., Eldin M.S.M., El-Meligy M.A. (2014). Physically crosslinked poly(vinyl alcohol)-hydroxyethyl starch blend hydrogel membranes: Synthesis and characterization for biomedical applications. Arab. J. Chem..

[49] Gadhave R.V., Mahanwar P.A., Gadekar P.T. (2019). Effect of glutaraldehyde on thermal and mechanical properties of starch and polyvinyl alcohol blends. Des. Monomers Polym..

[50] Pimentel G.C., Sederholm C.H. (1956). Correlation of Infrared Stretching Frequencies and Hydrogen Bond Distances in Crystals. J. Chem. Phys..

[51] Zaltariov M.F., Filip D., Varganici C.D., Macocinschi D. (2018). Atr-Ftir and Thermal Behavior Studies of New Hydrogel Formulations Based on Hydroxypropyl Methylcellulose/Poly(Acrilic Acid) Polymeric Blends. Cell. Chem. Technol..

[52] Holloway J.L., Lowman A.M., Palmese G.R. (2013). The role of crystallization and phase separation in the formation of physically cross-linked PVA hydrogels. Soft Matter.

[53] Zhang C., Liu X.H., Liu H., Wang Y.M., Guo Z.H., Liu C.T. (2019). Multi-walled carbon nanotube in a miscible PEO/PMMA blend: Thermal and rheological behavior. Polym. Test..

[54] Rodriguez-Rodriguez R., Garcia-Carvajal Z.Y., Jimenez-Palomar I., Jimenez-Avalos J.A., Espinosa-Andrews H. (2019). Development of gelatin/chitosan/PVA hydrogels: Thermal stability, water state, viscoelasticity, and cytotoxicity assays. J. Appl. Polym. Sci..

[55] Gomez I., Otazo E.M., Hernandez H., Rubio E., Varela J., Ramirez M., Barajas I., Gordillo A.J. (2015). Thermal degradation study of PVA derivative with pendant phenylthionecarbamate groups by DSC/TGA and GC/MS. Polym. Degrad. Stabil..

[56] Holland B.J., Hay J.N. (2001). The thermal degradation of poly(vinyl alcohol). Polymer.

[57] Shahbazi M., Rajabzadeh G., Ettelaie R., Rafe A. (2016). Kinetic study of kappa-carrageenan degradation and its impact on mechanical and structural properties of chitosan/kappa-carrageenan film. Carbohyd. Polym..

[58] Teodorescu M., Bercea M., Morariu S. (2018). Biomaterials of Poly(vinyl alcohol) and Natural Polymers. Polym. Rev..

[59] Fathi E., Atyabi N., Imani M., Alinejad Z. (2011). Physically crosslinked polyvinyl alcohol-dextran blend xerogels: Morphology and thermal behavior. Carbohyd. Polym..

[60] Salleh M.S.N., Nor N.N.M., Mohd N., Draman S.F.S. Water Resistance and Thermal Properties of Polyvinyl Alcohol-Starch Fiber Blend Film. Proceedings of the 6th International Advances in Applied Physics and Materials Science Congress & Exhibition (Apmas 2016).

[61] van de Velde F., Antipova A.S., Rollema H.S., Burova T.V., Grinberg N.V., Pereira L., Gilsenan P.M., Tromp R.H., Rudolph B., Grinberg V.Y. (2005). The structure of kappa/iota-hybrid carrageenans II. Coil-helix transition as a function of chain composition. Carbohyd. Res..

[62] Cha W.I., Hyon S.H., Ikada Y. (1993). Microstructure of Poly(Vinyl Alcohol) Hydrogels Investigated with Differential Scanning Calorimetry. Makromol. Chem..

[63] El-Zaher N.A., Osiris W.G. (2005). Thermal and structural properties of poly(vinyl alcohol) doped with hydroxypropyl cellulose. J. Appl. Polym. Sci..

[64] Stavropoulou A., Papadokostaki K.G., Sanopoulou M. (2004). Thermal properties of poly(vinyl alcohol)-solute blends studied by TMDSC. J. Appl. Polym. Sci..

[65] Braudo E.E., Muratalieva I.R., Plaschina I.G., Tolstoguzov V.B., Markovich I.S. (1991). Studies on the Mechanisms of Gelation of Kappa-Carrageenan and Agarose. Colloid. Polym. Sci..

[66] Stenner R., Matubayasi N., Shimizu S. (2016). Gelation of carrageenan: Effects of sugars and polyols. Food Hydrocolloid..

[67] Gunasekaran S., Wang T., Chai C.X. (2006). Swelling of pH-sensitive chitosan-poly(vinyl alcohol) hydrogels. J. Appl. Polym. Sci..

[68] Xiao C.M., Yang M.L. (2006). Controlled preparation of physical cross-linked starch-g-PVA hydrogel. Carbohyd. Polym..

[69] Imtiaz N., Niazi M.B.K., Fasim F., Khan B.A., Bano S.A., Shah G.M., Badshah M., Menaa F., Uzair B. (2019). Fabrication of an Original Transparent PVA/Gelatin Hydrogel: In Vitro Antimicrobial Activity against Skin Pathogens. Int. J. Polym. Sci..

[70] Zangi R., Engberts J.B.F.N. (2005). Physisorption of hydroxide ions from aqueous solution to a hydrophobic surface. J. Am. Chem. Soc..

[71] Necas J., Bartosikova L. (2013). Carrageenan: A review. Vet. Med. Czech..

[72] Malana M.A., Zohra R. (2013). The release behavior and kinetic evaluation of tramadol HCl from chemically cross linked Ter polymeric hydrogels. Daru.

[73] Singh B., Vashishtha M. (2008). Development of novel hydrogels by modification of sterculia gum through radiation cross-linking polymerization for use in drug delivery. Nucl. Instrum. Meth. B.

[74] Brazel C.S., Peppas N.A. (2000). Modeling of drug release from swellable polymers. Eur. J. Pharm. Biopharm..

[75] Hill D.J.T., Zainuddin Whittaker A.K. (2011). Water diffusion into radiation crosslinked PVA-PVP network hydrogels. Radiat. Phys. Chem..

[76] Gun’ko V.M., Savina I.N., Mikhalovsky S.V. (2017). Properties of Water Bound in Hydrogels. Gels (Basel).

[77] Ayawei N., Ebelegi A.N., Wankasi D. (2017). Modelling and Interpretation of Adsorption Isotherms. J. Chem. N. Y..

[78] Girods P., Dufour A., Fierro V., Rogaume Y., Rogaume C., Zoulalian A., Celzard A. (2009). Activated carbons prepared from wood particleboard wastes: Characterisation and phenol adsorption capacities. J. Hazard. Mater..

[79] Foo K.Y., Hameed B.H. (2010). Insights into the modeling of adsorption isotherm systems. Chem. Eng. J..

[80] Wu F.C., Liu B.L., Wu K.T., Tseng R.L. (2010). A new linear form analysis of Redlich-Peterson isotherm equation for the adsorptions of dyes. Chem. Eng. J..

[81] Yuan Z.Y., Wang J., Wang Y.M., Liu Q., Zhong Y.J., Wang Y., Li L., Lincoln S.F., Guo X.H. (2019). Preparation of a poly(acrylic acid) based hydrogel with fast adsorption rate and high adsorption capacity for the removal of cationic dyes. RSC Adv..

[82] Hosseinzadeh H. (2015). Synthesis of carrageenan/multi-walled carbon nanotube hybrid hydrogel nanocomposite for adsorption of crystal violet from aqueous solution. Pol. J. Chem. Technol..

[83] Mahdavinia G.R., Bazmizeynabad F., Seyyedi B. (2015). kappa-Carrageenan beads as new adsorbent to remove crystal violet dye from water: Adsorption kinetics and isotherm. Desalin. Water Treat..

[84] Kulkarni M.R., Anirudh T.R., Acharya A., Bhat P. (2017). Removal of Crystal Violet dye from aqueous solution using water hyacinth: Equilibrium, kinetics and thermodynamics study. Resour. Effic. Technol..

[85] Miyah Y., Lahrichi A., Idrissi M., Boujraf S., Taouda H., Zerrouq F. (2017). Assessment of adsorption kinetics for removal potential of Crystal Violet dye from aqueous solutions using Moroccan pyrophyllite. J. Assoc. Arab Univ. Basic Appl. Sci..

[86] Wu P., Cai Z., Jin H., Tang Y. (2019). Adsorption mechanisms of five bisphenol analogues on PVC microplastics. Sci. Total Environ..

